# Cauterisation of the Pharynx with Ammonia

**Published:** 1845-02

**Authors:** 


*Cauterisation of the Pharynx with Ammonia.*—M. Monneret, physician to the Hopital la Charité, has been trying the experiment of cauterising the pharynx with ammonia. In one case of bronchitis, accompanied by great dyspnœa, the relief was immediate. Others have repeated the experiment with satisfactory results. M. Rayer proposes to substitute cauterisation of the palate as less dangerous. The pharynx or palate is rapidly touched with liquid ammonia, diluted with one-third of water.—*Gazette des Hopitaux.*

